# Mechanisms of muscle insulin resistance and the cross‐talk with liver and adipose tissue

**DOI:** 10.14814/phy2.14607

**Published:** 2020-10-10

**Authors:** Simone C. da Silva Rosa, Nichole Nayak, Andrei Miguel Caymo, Joseph W. Gordon

**Affiliations:** ^1^ Department of Human Anatomy and Cell Science University of Manitoba Winnipeg Canada; ^2^ The Diabetes Research Envisioned and Accomplished in Manitoba (DREAM) Theme University of Manitoba Winnipeg Canada; ^3^ Children’s Hospital Research Institute of Manitoba (CHRIM) University of Manitoba Winnipeg Canada; ^4^ College of Nursing University of Manitoba Winnipeg Canada

**Keywords:** adipose tissue, insulin resistance, lipotoxicity, liver, mitochondrial dysfunction, skeletal muscle

## Abstract

Insulin resistance is a metabolic disorder affecting multiple tissues and is a precursor event to type 2 diabetes (T2D). As T2D affects over 425 million people globally, there is an imperative need for research into insulin resistance to better understand the underlying mechanisms. The proposed mechanisms involved in insulin resistance include both whole body aspects, such as inflammation and metabolic inflexibility; as well as cellular phenomena, such as lipotoxicity, ER stress, and mitochondrial dysfunction. Despite numerous studies emphasizing the role of lipotoxicity in the pathogenesis of insulin resistance, an understanding of the interplay between tissues and these proposed mechanisms is still emerging. Furthermore, the tissue‐specific and unique responses each of the three major insulin target tissues and how each interconnect to regulate the whole body insulin response has become a new priority in metabolic research. With an emphasis on skeletal muscle, this mini‐review highlights key similarities and differences in insulin signaling and resistance between different target‐tissues, and presents the latest findings related to how these tissues communicate to control whole body metabolism.

## Graphical abstract

Overview of insulin resistance mechanisms: the ability of the mitochondria to respond to metabolic disruptions is essential for healthy cellular bioenergetics, and interference with this process may prompt unregulated mitochondrial biogenesis and mitophagy, thus contributing to insulin resistance in major insulin target tissues.

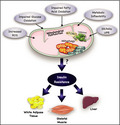

## 1 INSULIN DISCOVERY AND FUNCTION

### 1.1 Insulin overview

Frederick Banting and Charles Best are credited with the discovery of insulin in 1921 while working at the University of Toronto, later receiving a Nobel Prize in 1923. For nearly 100 years, the regulation of insulin secretion and its actions on peripheral tissues has been at the forefront of cell biology and physiological research. Arguably the most studied hormone in history, the study of insulin is embedded within most major cell biology discoveries, ranging from pro‐hormone production and trafficking, membrane biology, exocytosis, receptor tyrosine kinases, SH2 domains, glucose transporter (GLUT)4 trafficking, and the regulation of carbohydrate, lipid, and protein metabolism, to name a few. More recently, the study of mitochondrial function, autophagy, and mitophagy in the peripheral tissues has provided insight into how muscle, liver, and adipose tissues regulate their sensitivity to insulin function, and to how these key target tissues of insulin communicate with each other to control whole body metabolism.

In myofibers, hepatocytes, and adipocytes, insulin binds to receptors on the plasma membrane and coordinates anabolic responses to nutrient availability. Upon insulin binding to its receptor, a signaling cascade of events are activated, ultimately promoting glucose uptake, especially in muscle and adipose tissues which express high levels of the GLUT4 transporter. Additionally, insulin action impacts fatty acid, amino acid, and potassium uptake in muscle and fat tissues. If this system becomes disrupted, it gives rise to insulin resistance, which affects virtually every tissue in the body, but has a dominant effect on muscle, adipose, and liver tissues. Despite the growing body of literature highlighting various aspects of insulin signaling impairment leading to insulin resistance, the underlying molecular mechanisms are yet to be fully understood. In this review, we will summarize some of the critical mechanisms of insulin resistance in insulin target tissues, specifically highlighting skeletal muscle, but a comparison to liver and adipose tissue is also included.

### 1.2 Insulin receptors and substrates

The insulin receptor (INSR) is composed of both alpha (⍺) and beta (β) subunits, where in the β subunits, tyrosine phosphorylation is more specific to insulin binding, and this subunit is highly expressed in differentiated liver, muscle, and white adipose tissue (WAT) (Wei et al., 1995). The binding events are an essential step for INSR substrate recruitment and activation of downstream mitogenic and metabolic signals (Youngren, 2007). These signal activations are dependent on insulin concentrations. While induction of the metabolic response requires lower insulin amounts, the mitogenic response requires higher concentrations (Bedinger & Adams, 2015).

In all cell types, the activation of INSR is mediated upon the recruitment of phosphotyrosine‐binding scaffold proteins (PTB), initiating a cascade of cellular phosphorylation (Hubbard, 2013). The INSR receptor substrates include IRS1 and IRS2, Src homology collagen (Src), and adaptor protein (APS), with a pleckstrin homology (PH) and Src homology 2 (SH2) domain. Once phosphorylated, these substrates bind and activate kinases, mediating the initiation of insulin action in the cell (Youngren, 2007). The insulin receptor substrate (IRS) is the best‐described class of the INSR scaffold. Even though there are six IRS isoforms, IRS1 and IRS2 are the ones mediating most metabolic effects of INSR (Araki et al., 1994; Sun et al., 1991). The IRS contains an amino (NH2) and a carboxyl (COOH) terminal full of tyrosine and serine/threonine phosphorylation sites (White, 2012). IRS PTB domain bind to INSR pTyr972 to phosphorylate IRS tyrosine residues. After that, downstream signaling effectors are recruited to propagate and amplify insulin response (Hubbard, 2013). The S6 kinase (S6K) is the predominant inhibitor of insulin signaling, mediated by a negative feedback serine phosphorylation of IRS (Hsu et al., 2011). Furthermore, IRS phosphorylation is one of the major targets of stimuli during insulin resistance.

## 2 INSULIN RESISTANCE

Insulin resistance is characterized by a diminished response to insulin stimulation, resulting in the failure of target tissues to adequately dispose of blood glucose, inhibit lipolysis, stimulate glycogen synthesis, and inhibit hepatic glucose output (Petersen & Shulman, 2018). Traditionally viewed as a compensatory response, insulin secretion is enhanced leading to hyperinsulinemia. These defects may be reversible by weight loss, exercise, and proper nutritional diets; however, if left unopposed insulin resistance is a precursor event that likely contributes to β‐cell dysfunction. The most widely reported consequences of insulin resistance include the onset of type 2 diabetes (T2D), with corresponding fasting and postprandial hyperglycemia, elevated HbA1c, and nonalcoholic fatty liver disease (NAFLD), accompanied by fasting plasma hyperinsulinemia. However, more recent evidence using genetically engineered mouse models, have challenged the idea that insulin resistance is a direct cause of T2D, and instead posits that basal hyperinsulinemia caused by β‐cell gluco/lipotoxicity drives obesity and resistance in peripheral tissues (Mehran et al., 2012; Templeman et al., 2015). Collectively, these insights support the notion of a feed‐forward system whereby insulin resistance stimulates hyperinsulinemia, and hyperinsulinemia worsens obesity and insulin resistance until β‐cells fail, marking the onset of T2D.

### 2.1 Lipotoxicity

**FIGURE 1:**
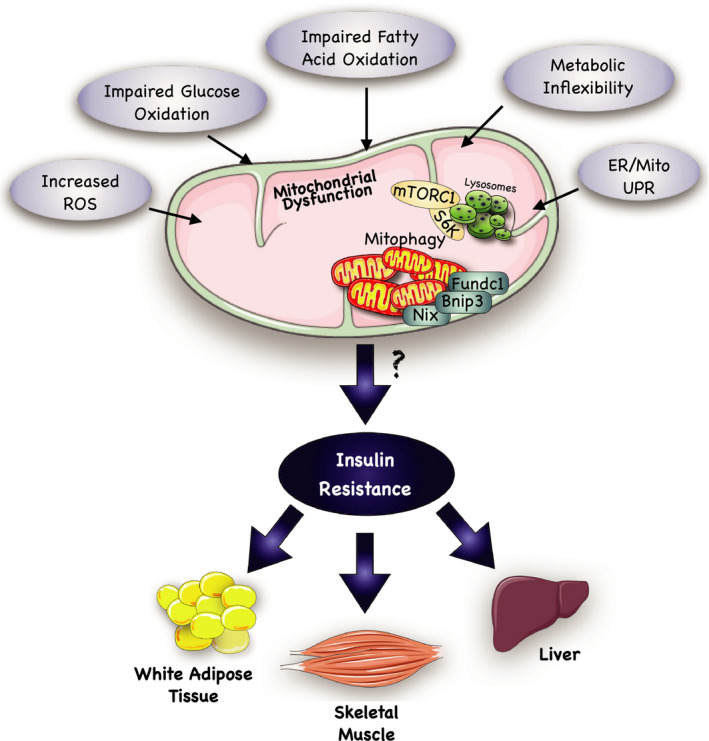
Overview of possible mechanisms leading to insulin resistance. Impaired mitochondrial functioning is a suggested mechanism through which insulin resistance develops. Several factors have been implicated in the development of mitochondrial dysfunction. Some of the most reported factors include: (a) an increased production of reactive oxidative species (ROS). ROSs are necessary by‐products of mitochondrial energy metabolism; however, if their production is not adequately coupled by intracellular antioxidants, then oxidative damage to mitochondrial DNA (mtDNA) can occur. (b) Impaired glucose oxidation and fatty acid oxidation may result from metabolic inflexibility, which is commonly described as an inability to adapt fuel oxidation to fuel availability. Ultimately, these events may lead to both the development of ROS and ectopic lipid accumulation, resulting in mitochondrial damage and removal through mitophagy pathways. Further investigation on the genes involved in mitophagy (i.e., Bnip3, Nix, Fundc1) may allow the discovery of pathways that explain the correlation between mitochondrial dysfunction and insulin resistance in adipose, hepatic, and skeletal muscle tissue. (c) Endoplasmic Reticulum (ER) stress is also involved in the pathogenesis of mitochondrial dysfunction. Lipotoxicity and glucotoxicity can induce ER stress, which may trigger an adaptive signaling pathway, known as the unfolded protein response (UPR). If ER stress fails to be relieved by this UPR, then this can lead to the provocation of both mitochondrial dependent and independent cell death pathways. Together, the ability of the mitochondria to respond to metabolic disruptions is essential for healthy cellular bioenergetics, and interference with this process may prompt unregulated mitochondrial biogenesis and mitophagy, thus contributing to insulin resistance. This figure was created using Servier Medical Art (available at https://smart.servier.com/) and PAGES software.

Among the leading etiological factors in the pathogenesis of insulin resistance is the effect of lipotoxicity. Lipotoxicity is a type of cellular stress induced by the accumulation of lipid intermediates such as diacylglycerols (DAGs), ceramides, and triglycerides that facilitate the development of insulin resistssance in muscle, liver, and adipose tissue (Erion & Shulman, 2010; Gordon et al., 2015; Petersen & Shulman, 2018; Samuel & Shulman, 2016). Moreover in skeletal muscle, the overabundance of fatty acid intermediates impedes insulin signaling via the reduction of GLUT4 transporters on the myocyte membrane surface (Chavez & Summers, 2003; Itani et al., 2002; Montell et al., 2001). In other tissues such as the liver, intrahepatic triglycerides (IHTG) accumulation, and DAG‐PKC‐ε axis have been implicated in the pathogenesis of hepatic insulin resistance (Marchesini et al., 1999). As for adipose tissue, it has been theorized that an increase in lipolysis, governed by a similar cascade found in skeletal and liver tissue as seen in Figure 1, is the proximal cause of insulin resistance at this site (Morigny et al., 2016).

### 2.2 Metabolic inflexibility

Metabolic flexibility is described as the ability of an organism to adapt fuel oxidation to fuel availability (Goodpaster & Sparks, 2017). Consequently, metabolic inflexibility is characterized by impaired fuel switching and energy dysregulation, concepts that are both closely associated with insulin resistance and cardiometabolic disease (Muoio, 2014). Diminished fuel switching capacity can result in the accumulation of intramyocellular lipid (IMCL), as well as DAG‐PKCθ activation and impairment of proximal insulin signaling pathways (Rahimi et al., 2014). The latter ultimately impairs insulin signaling through different mechanisms, either increased serine phosphorylation of IRS1 at Ser‐1101 and/or reduced serine phosphorylation of PKB/Akt (Morino et al., 2006; Summers & Nelson, 2005).

In response to an excess of fatty acid availability, fatty acid transporters may limit cellular and mitochondrial fatty acid uptake, thereby reducing fat oxidation and increasing the accumulation of lipotoxic lipid metabolites, contributing to the onset of insulin resistance (Corpeleijn et al., 2009). Additionally, myotubes from insulin‐sensitive subjects have been reported to be more adaptive to fatty acid exposure in vitro (Perreault et al., 2018). Furthermore, palmitate oxidation has also been shown to be lower in myotubes derived from T2D versus matched nondiabetic controls (Gaster, 2007; Gaster et al., 2004). Ultimately, defects in fuel switching can intensify with impaired mitochondrial content and/or function, further contributing to insulin resistance and mitochondrial dysfunction (Figure 1).

## 3 SKELETAL MUSCLE

### 3.1 Insulin signaling

The skeletal muscle accounts for approximately 80% of postprandial glucose disposal in humans, and proper insulin action is imperative to maintain glucose homeostasis (DeFronzo & Tripathy, 2009; Shulman et al., 1990; Thiebaud et al., 1982). The primary role of glucose in the skeletal muscle is to promote glycolysis or glycogen synthesis, where the latter represents 75% of all glucose disposal (DeFronzo & Tripathy, 2009).

**FIGURE 2:**
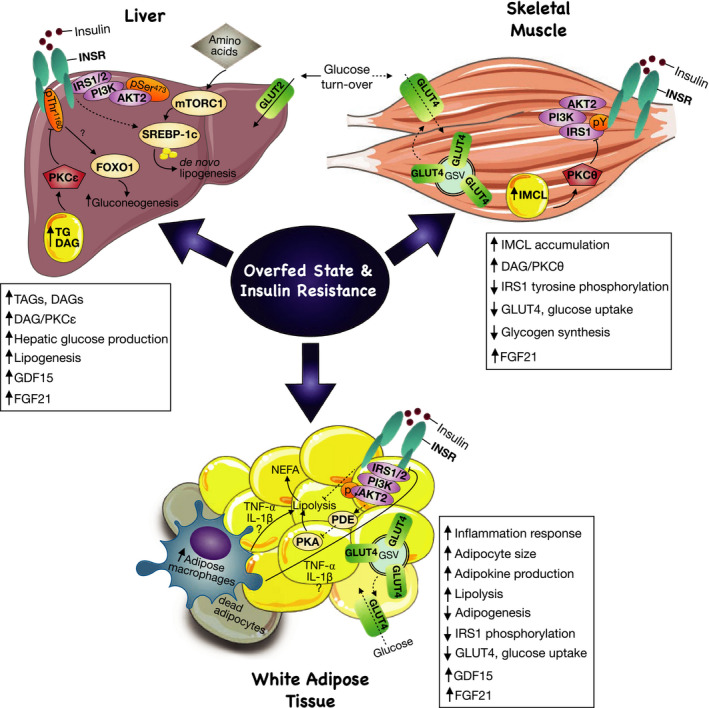
Overfed state and insulin resistance as central regulators in tissue‐crosstalk. An increase in adipokines causes augmentation in the inflammatory response and recruitment of macrophages and cytokines around dead adipocytes to discard them. The inflammatory mediators, tumor necrosis factor (TNF)‐⍺ or interleukin‐1 (IL‐1)‐β increase lipolysis and inhibit insulin receptor (INSR), therefore, impairing insulin signaling. Lipolysis is activated by protein kinase A (PKA) signaling, and, in a fed state, insulin activates Akt2 signaling, which via unknown mechanisms activates phosphodiesterase 3 (PDE3) and inhibits PKA in order to suppress lipolysis. However, during adipose tissue insulin resistance, there is a decrease in Akt2 phosphorylation, contributing to sustained lipolysis activation. As a result, non‐esterified fatty acid (NEFA) production and circulating fatty acids are increased, which are uptake by the liver and muscle, contributing to ectopic lipid accumulation in both tissues. Intrahepatic lipid accumulation triggers activation of the diacylglycerol (DAG)/protein kinase C (PKC)ε axis. PKCε directly phosphorylates and inhibits insulin receptor (INSR) at Thr1160, impairing insulin signaling. As a result, gluconeogenesis is increased, as insulin suppression of gluconeogenesis mediator, forkhead box protein 01 (FOXO1), is impaired. Furthermore, in a chronic overnutrition state, de novo lipogenesis (DNL) is increased, concurrent with its mediators mTORC1 and SREBP‐1c. Even though activation of SREBP‐1c by INSR is impaired in hepatic insulin resistance, other inputs such as amino‐acid mediated mTORC1 activation can increase the lipogenic flux. Additionally, a chronic increase in lipolysis contributes to IMCL accumulation and lipid‐induced insulin resistance. In contrast to the liver, in the skeletal muscle, lipid‐induced insulin resistance is initiated upon activation of the DAG‐PKCθ axis and inhibition of both phosphoinositide‐3 kinase (PI3k) and phosphorylation of IRS1. As a result, insulin signaling is impaired, preventing glucose transporter (GLUT)4 translocation to the plasma membrane and glucose uptake. As a compensatory mechanism during an overfed state, FGF21 plays a role as a hepatokine, adipokine, and myokine controlling insulin sensitivity in insulin‐resistant animals. Similarly, GFD15 is selectively upregulated as a stress response mechanism during high‐fat feeding, especially in liver and adipose tissue. This figure was created using Servier Medical Art (available at https://smart.servier.com/) and PAGES software.

Elevated levels of blood glucose trigger pancreatic insulin release, which subsequently binds to INSR to promote glucose uptake and glycogen storage. Moreover INSR stimulation triggers a phosphorylation‐dephosphorylation cascade that is mediated by various kinases such as S6 kinase (S6K), protein kinase B (Akt), 3‐phosphoinositide‐dependent protein kinase 1 (PDK1), and isoforms of PKC (Boucher et al., 2014). These proteins function to regulate numerous pathways in the skeletal muscle that contribute to glucose metabolism (Boucher et al., 2014). As shown in Figure 2, one signaling pathway involves the translocation of GLUT4 containing storage vesicles (GSVs) to the plasma membrane, which is regulated by Akt2 (DeFronzo & Tripathy, 2009; Taniguchi et al., 2006). Other downstream effects result in an increase in G6P (glucose‐6‐phosphate), dephosphorylation of glycogen metabolic proteins, and glycogen synthesis (DeFronzo & Tripathy, 2009). Therefore, in order to sustain normal insulin‐stimulated glucose uptake in the muscle, the IRS1/PI3K/Akt pathway has to be maintained (DeFronzo & Tripathy, 2009).

Tyrosine phosphorylation of IRS mediates PI3K recruitment and insulin stimulated glucose uptake. Conversely, serine and threonine phosphorylation of IRS promote the opposite effects (Pederson et al., 2001). Continuous exposure to high insulin levels were found to induce phosphorylation of either serine or threonine, which consequently inhibited IRS1 and decreased GLUT4 translocation to the plasma membrane (Pederson et al., 2001). Skeletal muscle uses IRS1 as the primary substrate for INSR during insulin‐mediated glucose metabolism (Boucher et al., 2014). Although IRS2 is also expressed, it is unnecessary for insulin‐stimulated glucose transport in the muscle (Higaki et al., 1999). In the presence of high amounts of phosphatidylinositol (Aguirre et al., 2000; Akhtar et al., 2019; Albers et al., 2015)‐trisphosphate (PIP3) in the cellular membrane, kinases with a PH domain such as PDK1 and Akt are recruited (Gao et al., 2011). While both Akt1 and Akt2 are present in skeletal muscle, Akt2 is more critical for insulin‐stimulated glucose uptake. Studies on mice have indicated that Akt2 knockouts are extremely glucose intolerant (Cho, et al., 2001), whereas Akt1 knockouts exhibit normal glucose tolerance, along with growth defects (Cho et al., 2001b).

PI3K has been demonstrated to regulate GLUT4 translocation (Hausdorff et al., 1999). It is mediated by Akt2 phosphorylation and the activity of a Rho family of small guanosine triphosphatases (GTPase) Ras‐related C3 botulinum toxin substrate 1 (RAC1) (Gonzalez & McGraw, 2009; Zeigerer et al., 2004). Studies on muscle‐specific RAC1 knockouts in mice have indicated that Rho GTPases may be involved in insulin‐stimulated glucose uptake regulation in the muscle via PI3K‐dependent signaling (Wu et al., 2019). Further, Sylow et al. (2013) observed that even in the presence of activated Akt, these knockout mutations resulted in impaired insulin‐stimulated glucose uptake. Despite its relevance to insulin‐stimulated glucose uptake signaling, the precise mechanisms of RAC1 induced GLUT4 translocation is not entirely known.

In summary, GLUT4 translocation to the plasma membrane in a fed state is an important step in normal blood glucose disposal in the skeletal muscle and is characteristic of normal insulin physiology. However, the failure of this event in response to insulin indicates an early stage of insulin resistance and T2D (Leto & Saltiel, 2012).

### 3.2 Insulin resistance

One of the earliest theories proposed to explain the mechanisms of muscle insulin resistance was postulated by Randle and his colleagues, in the 1960s. They concluded that an acute increase in muscle fatty acid oxidation leads to the accumulation of citrate, which inhibits phosphofructokinase (PFK), a pivotal enzyme in glycolysis (Randle et al., 1963). This results in the impairment of glucose utilization (Randle et al., 1963). Other studies have further elaborated that reduced insulin‐stimulated muscle glycogen synthesis and glucose oxidation may drive chronic insulin resistance (Shulman et al., 1990). However, human studies examining GLUT4 function have suggested that failed INSR signaling cascade activation and impaired GLUT4 translocation is the primary defect in skeletal muscle insulin resistance (Garvey et al., 1998). Additionally, in T2D patients, tyrosine phosphorylation of IRS1 was reported to be severely impaired as a consequence of hyperglycemia (Abdul‐Ghani & DeFronzo, 2010). Therefore, defects at the proximal level of insulin signaling that involve INSR, IRS1, PI3K, and Akt pathways are more evident in skeletal muscle insulin resistance, resulting in a decrease in insulin‐stimulated glucose uptake (Figure 2).

Modern theories argue that skeletal muscle lipid exposure is one of the leading causes of muscle insulin resistance. The mechanism of lipid‐induced insulin resistance linking DAGs, ceramides and other species have been extensively investigated (Gassaway et al., 2018; Muoio & Newgard, 2008; Petersen & Shulman, 2018; Shulman, 2000). Animals treated with a high‐fat diet and lipid infusion increased muscle DAG, resulting in the activation of PKCθ (Yu et al., 2002). Similarly, lipid infusions in humans resulted in an increase in DAG and PKCθ signaling, which impairs tyrosine phosphorylation of IRS1 activation (Szendroedi et al., 2014; Yu et al., 2002). As IRS1 is the primary activator of PKB/Akt, inhibition of this pathway blocks insulin signaling cascade events preventing insulin‐stimulated glucose uptake in the skeletal muscle (Figure 2). In other studies, acute induction of muscle insulin resistance increased DAG content, PKCθ activation, and increased phosphorylation of IRS1 serine 1,101, concurrent with inhibition of IRS1 and Akt2 phosphorylation. However, adipokines, ceramides, and acylcarnitine content alterations were not associated with insulin resistance in this study (Szendroedi et al., 2014).

In both skeletal muscle and liver, activation of the novel PKC (nPKC) isoforms have been consistently observed (Figure 2). Yu and colleagues (2002) suggested a link between increased DAG content and sustained activation of the PKCθ. Moreover within muscle tissue of high‐fat fed rodents, or in cultured myocytes exposed to palmitate, the expression of a smaller molecular weight catalytically active PKCδ fragment is substantially elevated to sustain heightened PKC activity (Mughal et al., 2015). In addition, infusion studies in rodents using lipid emulsions or glycerol, have observed nPKC‐mediated insulin resistance results with chronic IMCL accumulation (Samuel & Shulman, 2016; Yu et al., 2002). Specifically in skeletal muscle, T2D patients present with both PKCθ (Szendroedi et al., 2014), and PKCε (Perreault et al., 2018) isoforms increased, compared to controls. Interestingly, a study conducted on endurance athletes found that IMCL was accumulated in athletes who did not have T2D, a phenomenon termed the “Athlete's Paradox” (Daemen et al., 2018). It was discovered that T2D patients and endurance athletes store lipid droplets differently. T2D patients have lipid droplets localized in the subsarcolemmal region of type II muscle fibers while the athlete's lipid droplets were localized in the myofibrillar region of type I muscle fibers. This finding suggests the importance of lipid droplet morphology and storage in the consequent pathogenesis of insulin resistance (Daemen et al., 2018).

Another proposed mechanism involves the presence of branched chain amino acids (BCAAs), as serum BCAAs were shown to be elevated in obese T2D patients (Felig et al., 1974). Similarly, animal and human studies have shown that the infusion of amino acids caused impairment of skeletal muscle glucose uptake (Krebs et al., 2002; Um et al., 2006). Recently, pharmacological enhancement of BCAA catabolic activity has been shown to improve insulin resistance and hyperglycemia, at least in an animal model (Zhou et al., 2019). The mechanisms of BCAA are not clear, but it has been linked to lipotoxicity leading to insulin resistance (Sun & Wang, 2019). Other studies have shown that high amounts of BCAA can interfere with insulin signaling via activation of mTOR and S6K1 in a PI3K dependent manner (Um et al., 2006). Tremblay et al. (2007) identified in an in vitro model that S6K1 directly phosphorylates IRS1 Ser‐1101 resulting in suppression of IRS1 tyrosine and Akt phosphorylation, and further, insulin resistance (Tremblay et al., 2007).

Skeletal muscle is a heterogeneous tissue with different muscle fibers. Studies suggest that insulin‐stimulated glucose metabolism is different according to each muscle fiber type (Schiaffino & Reggiani, 2011; Talbot & Maves, 2016). On a physiological level, slow‐twitch (type I) fibers had higher amounts of GLUT4 and hexokinase II, among others, but lower Akt2, TBC1D4, and TBC1D1 amounts compared to type 2 fibers (Albers et al., 2015). These studies concluded that type I fibers have better glucose‐handling capacity, but similar insulin phospho‐regulation sensitivity (Albers et al., 2015). Furthermore, in other studies, obese and T2D patients presented with a lower proportion of type I fibers, which is rich in mitochondria, compared to type II fibers, concurrent with reduced oxidative metabolism (Oberbach et al., 2006).

In obesity and insulin resistance, the skeletal muscle capillary network is compromised, impairing insulin‐mediated capillary recruitment. A study by Umek et al. (2019) investigated the possibility that the anatomical changes in the capillary network could be linked to fiber‐type specific differences. Capillary density was found to be increased in small muscle fibers (type I) compared to large fibers (type II) and is attributed to increased capillarization selectiveness towards more insulin‐sensitive oxidative muscle fibers (Umek et al., 2019). Their findings suggest that the selective increase in capillarization surrounding more insulin‐sensitive oxidative muscle fibers act to alleviate obesity‐related insulin resistance (Umek et al., 2019). Obese insulin‐resistant humans and mice present muscle fiber type transformation, which provides a possible mechanism related to impaired glucose metabolism and T2D. Although intriguing in the context of diabetic microangiopathy, further studies are required to more fully appreciate these observations (Albers et al., 2015; Umek et al., 2019).

Recent theories argue that excessive β‐oxidation and mitochondria acylcarnitine accumulation is linked to the development of muscle insulin resistance (Koves et al., 2008). Koves et al. (2008) found that concurrent with the upregulation in β‐oxidation there was a decrease in tricarboxylic acid (TCA) intermediates. Furthermore, in this imbalanced environment of excessive β‐oxidation, the mitochondria becomes more susceptible to accumulation of acyl‐CoAs and acylcarnitine, possibly contributing to mitochondrial failure.

Gene expression arrays performed on human muscle biopsies found that a series of genes involved in oxidative metabolism were downregulated in T2D patients (Hesselink et al., 2016). For instance, PGC‐1α, responsible to promote mitochondrial biogenesis, is decreased in T2D along with several of its target genes. This observation has been proposed as an explanation for the decrease in muscle oxidative capacity observed in T2D (Mootha et al., 2003; Patti et al., 2003). With a decrease in mitochondrial function, fatty acids compete with glucose for oxidative degradation (Randle et al., 1963), resulting in metabolic inflexibility (Figure 1). This inability to switch fuel oxidation upon nutrient availability likely contributes to insulin resistance and IMCL accumulation (Chow et al., 2017). The IMCL accumulation and insulin resistance in the skeletal muscle emphasizes the role of dysfunctional mitochondrial in contributing to ectopic lipid accumulation in both elder (Petersen et al., 2003) and younger subjects (Petersen et al., 2009).

Finally, recent evidence suggests that inflammation occurs in skeletal muscle in obesity, characterized by increased immune cell infiltration and proinflammatory activation in intermyocellular tissue (Wu & Ballantyne, 2017). Increased levels of cytokines such as TNF‐α and IL‐1β activate the PKC, JNK, and IKK/NF‐κB pathways in myocytes. As a result, this may impair insulin signaling via serine or threonine phosphorylation, which disrupts insulin‐stimulated tyrosine phosphorylation of IR or IRS.

### 3.3 Mitochondrial dysfunction

Mitochondria are dynamic organelles that adapt to metabolic perturbations by undergoing fusion and fission cycles, rearranging electron transport chain complexes into supercomplexes, and biogenesis via peroxisome proliferator‐activated receptor γ co‐activator 1α (PGC 1α) (Sergi et al., 2019). These normal processes, however, are dysregulated in individuals with T2D and insulin resistance (Jeong‐a et al., 2008; Lowell & Shulman, 2005). Mechanistic studies have shown that lipotoxicity prompts excessive mitochondrial fission via DRP1 activation, resulting in impaired insulin‐stimulated glucose uptake (Jheng et al., 2012). Another presumed mechanism connecting mitochondrial dysfunction to insulin resistance is the generation of reactive oxygen species (ROSs) by mitochondria (Figure 1). Notably, oxidative stress occurs when ROS production overwhelms cellular antioxidant capacity. In addition to ROSs ability to induce oxidative damage to nuclear and mitochondrial DNA, lipids and protein, they are also signaling molecules that can directly induce insulin resistance (Schieber & Chandel, 2014). The induced oxidative damage caused by the ROSs consequently triggers the removal of damaged mitochondria by mitophagy (Wei et al., 2014). The resultant decrease in mitochondrial function and density compromises overall cellular oxidative capacity, further contributing to ectopic lipid accumulation and onset of insulin resistance (Anderson et al., 2009; Montgomery & Turner, 2014). Lee et al. (2017) investigated this hypothesis in mice by overexpressing a mitochondrial‐target catalase (MCAT) and fed them with a high‐fat diet. These animals were protected from energy imbalance, as well as from DAG accumulation and PKCθ activation. However, it is debatable whether or not muscle mitochondrial ROS production directly contributes to lipid‐induced insulin resistance.

### 3.4 Mitochondrial dynamics and permeability transition

Mitochondrial dynamics, which includes cycles of fission and fusion, is necessary for appropriately maintaining the mitochondria's shape, size and distribution in response to changing physiologic conditions (Yu & Pekkurnaz, 2018). For example, in the case of an absolute or relative drop in ATP, there is a shift toward mitochondrial fusion (Hesselink et al., 2016). Furthermore, mitochondrial fusion enables content mixing within the mitochondrial population, thereby preventing the loss of essential components (Westermann, 1817). Mitochondrial fission is also required to replenish the mitochondrial network (Youle & Karbowski, 2005), as it enables the removal of damaged mitochondria through mitophagy (Ding & Yin, 2012; Youle & Bliek, 2012). However, aberrant mitochondrial fission can lead to mitochondrial dysfunction and insulin resistance in skeletal muscle (Jheng et al., 2012).

Dysregulated mitochondrial fission is often associated with more severe mitochondrial dysfunction as this morphological state predominates during elevated stress levels and cell death (Jheng et al., 2012). Furthermore, increased mitochondrial fission and subsequent mitochondrial fragmentation have been associated with increased ROS production, mitochondrial depolarization, impaired ATP production, and decreased insulin‐dependent glucose uptake in C2C12 murine cell line (Kim et al., 2000), as well as increased mitochondrial ROS and impaired insulin signaling (Lin et al., 2018). A shift towards fission also negatively impacts fatty acid β‐oxidation (Jheng et al., 2012), which has been described as an important metabolic defect in insulin resistance (Koves et al., 2008). In support of this, a shift toward fusion has been reported to increase fatty acid consumption (Lionetti et al., 2014), presumably averting lipotoxicity.

Mitochondrial dynamics can also describe mitochondria‐organelle interactions such as the ER, peroxisomes, and nucleus (Xia et al., 2019). In particular, the dysregulation of the interactions between mitochondria and the ER has been stated to be implicated in the pathogenesis of muscle insulin resistance (Tubbs et al., 2018). Mitochondria‐ER contact points, also known as mitochondria‐associated ER membranes (MAMs), are the sites where Ca2+, lipid and metabolite exchange occur, thus representing critical points of interaction for the regulation of oxidative metabolism (Theurey & Rieusset, 2017). Additionally, studies have shown that the disruption of the MAMs in the liver promotes insulin resistance (Tubbs et al., 2014). Also, studies have shown that an increase in MAMs results in the accumulation of Ca2+ within the mitochondria, leading to compromised mitochondrial oxidative capacity, an increase in ROS production and impeded insulin signaling (Arruda et al., 2014). Subsequently, MAM formation appears to be an important regulator of mitochondrial function and insulin sensitivity.

Another proposed mechanism offering a causal link between mitochondrial dysfunction and insulin resistance involves the mitochondrial permeability transition pore (mPTP) protein complex. In particular, Taddeo et al. (2014) showed that genetic deletion of whole body Cyclophilin D, a mPTP gatekeeper protein, protected mice from diet‐induced glucose intolerance and increased glucose uptake specifically in skeletal muscle. Interestingly, the improved glucose tolerance was only associated with glucose uptake in the skeletal muscle, and the effects did not transfer to adipose and liver tissues. Furthermore, the protective effects of mPTP inhibition on insulin sensitivity did not involve changes to the insulin signaling pathway but rather, it occurred via an unmapped mechanism preventing the formation of GLUT4 vesicles (Taddeo et al., 2014), a phenomenon described by other studies (Tsuchiya et al., 2010).

Collectively, the ability of the mitochondria to dynamically respond to metabolic perturbations is essential for healthy cellular bioenergetics, and interference with this delicate process may prompt unregulated mitochondrial biogenesis and mitophagy, thus contributing to insulin resistance (Jheng et al., 2012).

### 3.5 Autophagy

In response to the energetic and metabolic demands during periods of cellular stress, cells undergo more frequent autophagy and induce catabolic processes (Feng et al., 2014; He & Klionsky, 2009; Kroemer et al., 2010; Mizushima, 2018; Mizushima & Levine, 2010). As the name implies, autophagy is a self‐consuming process that functions to mediate levels of various proteins in cells within different environments. This self‐digesting mechanism is imperative in the removal of damaged organelles and proteins by the lysosome. Mammalian autophagy has been identified to primarily involve numerous Atg proteins, autophagy mediators, and conjugation systems that allow for the formation of the autophagosome, which encapsulates the cargo to later be degraded by the lysosomes (Badadani, 2012; Kroemer et al., 2010; Yang & Klionsky, 2010).

Autophagy was initially posited to be a cellular response to starvation or nutrient deprivation; however, some studies have suggested that autophagy may potentially play a role in preventing insulin resistance in the fed state. Conversely, other studies have argued that autophagy actually contributes to the pathogenesis of insulin resistance (Rocha et al., 2020). The Bcl‐2‐Beclin‐1 complex is an important mediator of autophagy, where phosphorylation of Bcl‐2 can release Beclin‐1 from this complex and ultimately leads to the induction of autophagy (Pattingre et al., 2005). Intriguingly, He and colleagues (2012) found that transgenic mice expressing a Bcl‐2 mutant that could not inhibit Beclin‐1 correlated with a decrease in insulin sensitivity and GLUT4 translocation to the membrane surface. The authors concluded that the onset of acute exercise can contribute to disruptions in the coupling between the Bc1‐2‐Beclin‐1 complex, which consequently leads to increased autophagy in the skeletal muscle. Interestingly, Palikaras et al. (2018) elucidated a mechanism by which selective mitochondrial autophagy (mitophagy) occurs involving PINK1‐Parkin and LC3 interacting regions (LIR) to which the latter can serve as a receptor to induce mitochondrial autophagy.

### 3.6 Mitophagy

Alterations in mitochondrial dynamics and elevated levels of mitophagy have been linked to the onset of insulin resistance in T2D by multiple studies (Montgomery & Turner, 2014; Rocha et al., 2020; Rovira‐Llopis et al., 2017). Mitophagy (also known as selective autophagy of the mitochondria) is the process where dysfunctional mitochondria are degraded in a form of organelle turn‐over. One of the most well‐characterized pathways is the PINK1‐Parkin‐mediated mitophagy (Palikaras et al., 2018), stimulated by a decrease in mitochondrial membrane potential and associated with ROS elevations (Xiao et al., 2017). Upon mitochondrial depolarization, the PINK1 kinase accumulates on the outer mitochondrial membrane (OMM) and initiates recruitment of the E3 ubiquitin ligase Parkin. Parkin mediates the ubiquitination of several OMM proteins, leading to the recruitment and degradation of proteases and autophagosomes (Chan et al., 2011; Ordureau et al., 2018; Sarraf et al., 2013). In a human study, T2D patients presented a decrease in various mitophagy‐related genes, including PINK1; furthermore, mutations in PINK1 have also been associated with T2D in humans (Bhansali et al., 2017). Conversely, in healthy endurance‐trained runners, key mitophagy markers such as PINK1 and Drp1 were enhanced in skeletal muscle, and high‐fat meals had no influence over these markers (Tarpey et al., 2017).

Besides the PINK1‐Parkin ubiquitin‐dependent mitophagy mechanism, mitochondrial proteins also act as mitophagy receptors and target dysfunctional mitochondria for degradation by autophagosomes (Palikaras et al., 2018). The mitophagy receptors, such as BCL2L13, FKBP8, Fundc1, Bnip3, and Nix, initiate mitophagy via direct interaction with ATG8 proteins, such as LC3 and GABARAP, on the autophagosome membrane through their LIR motifs (Gatica et al., 2018). Fundc1 interacts with the ER calnexin proteins and recruits mitochondrial fission protein DRP1 to activate mitochondrial fission (Palikaras et al., 2018). Mitophagy initiated by hypoxia increases the expression of Fundc1, which interacts with and recruits LC3 proteins to dysfunctional mitochondria. A recent study showed that Fundc1 deletion in skeletal muscle resulted in impaired mitochondrial energetics due to LC3‐mediated mitophagy defect (Fu et al., 2018). However, in spite of the reduced muscle mitochondrial energetics and exercise capacity, these animals interestingly were protected against obesity and insulin resistance elicited by high‐fat (HF) feeding (Fu et al., 2018).

Like Fundc1, Bnip3, and Nix are also hypoxia‐inducible and regulators of mitophagy. Metabolic stresses such as lipotoxicity, hypoxia, and starvation, can all induce Bnip3/Nix mediated mitophagy (Glick et al., 2012; Moreira et al., 2017). Recent studies have demonstrated that Bnip3, Nix and Fundc1 mediated mitophagy plays an important role in the treatment of lipid metabolism and various hepatic dysfunctions (Chao et al., 2018; Glick et al., 2012; Li et al., 2019; Williams & Ding, 2015). Furthermore, Nix has also been previously demonstrated to become elevated in insulin‐resistant rodents (Mughal et al., 2015). In subsequent studies by our group, we have observed that mitophagy is linked to Nix accumulation in lipotoxic environments leading to impaired insulin signaling via activation of mTOR‐p70S6 kinase and inhibition of IRS1 (da Silva Rosa et al., 2020).

We demonstrated that Nix respond to lipotoxicity in order to clear damaged mitochondria through mitophagy. In turn, it protects the myocyte against nutrient storage stress via activation of mTOR‐dependent desensitization of insulin signaling, via phosphorylation of serine 1,101 of IRS1. Nix induced ER calcium release concurrent DRP1 activation, along with increased levels of mitophagy (da Silva Rosa et al., 2020). As previously described, DRP1 is as an important mediator of mitochondrial fission and mitophagy (Gandhi & Perry, 2020). Consistent with the results of Fu et al. (2018), who showed that deletion of the mitophagy receptor Fundc1 protects against HF feeding, and we observed that Nix is the most abundantly elevated mitophagy receptor in soleus muscle followed by HF feeding. Furthermore, we highlight a link between Nix‐induced mTOR activation and recruitment of lysosomal small GTPases such as Rheb to the mitochondria, to initiate mitophagy. This phenomenon was demonstrated to be dependent on phosphatidic acid availability (da Silva Rosa et al., 2020), which is also an important modulator of mitochondrial dynamics (Hornberger et al., 2006). In a cellular model, we demonstrated that knockdown of the mitochondrial phospholipase‐D, an enzyme responsible for converting cardiolipin to phosphatidic acid, prevented Nix‐induced mTOR‐S6K activation. Furthermore, Nix‐induced mitophagy and impaired insulin signaling could be reversed by direct phosphorylation of Nix at Serine 212 by PKA activating agents, such as clenbuterol and phosphodiesterase‐4 inhibitors (da Silva Rosa et al., 2020). This novel target may represent a future therapeutic strategy to circumvent the mitochondrial defects observed in muscle insulin resistance.

Collectively, these findings suggest an important mechanism linking excessive muscle mitochondrial turn‐over to impaired insulin‐stimulated glucose uptake. Nevertheless, the role of mitophagy in muscle insulin resistance and T2D is an emerging field, and its role and molecular mechanisms in these pathological processes require further in vivo experimentation (Figure 1).

### 3.7 ER stress

The mechanisms of lipid‐induced insulin resistance have been well described as an underlying cause of obesity‐associated insulin resistance. However, an overload of other nutrients may also be implicated in the etiology of insulin resistance (Villalobos‐Labra et al., 2019), and diabetes (White et al., 2020), for instance, the unfolded protein response (UPR), activated by ER stress (Villalobos‐Labra et al., 2019). The main role of the UPR is to promote an adaptive cellular response that alleviates ER stress through different mechanisms, such as the inhibition of protein synthesis and the enhancement of protein folding and degradation (Vincenz‐Donnelly & Hipp, 2017). The major arms of UPR activation in mammals are mediated by three ER transmembrane stress sensors: PKR‐like ER kinase (PERK), inositol requiring enzyme 1 (IRE‐1), and activating transcription factor 6 (ATF6) (Adams et al., 2019).

Although the precise role of ER stress in muscle insulin resistance remains uncertain, there are two mechanisms proposed in the contexts of obesity and T2D that are well supported by the literature. The first mechanism is the activation of the c‐Jun N‐terminal kinase (JNK1) pathway via IRE‐1, resulting in inhibitory serine phosphorylation of IRS‐1 (Aguirre et al., 2000; Solinas & Becattini, 2016). Moreover JNK was proposed to induce insulin resistance in obesity via four different mechanisms, including direct inhibition of IRS1 phosphorylation, induction of metabolic inflammation, increased adipogenesis and metabolic efficiency, and negative regulation of the PPARα‐FGF21 axis (Solinas & Becattini, 2016). The second mechanism linking ER stress, obesity and diabetes‐induced insulin resistance involves activation of the PERK/eIF2/ATF3 signaling pathway (Ohoka et al., 2005). Activation of this pathway leads to increased expression of the tribbles‐like protein 3 (TRB3). The TRB3 is an important pseudokinase that highly contributes to insulin resistance by inhibition of Akt activity (Ozcan et al., 2013).

## 4 LIVER

### 4.1 Insulin signaling

In the liver, insulin signaling is initiated by INSR trans‐autophosphorylation, activation, and recruitment of scaffold signaling proteins (Mugabo & Lim, 2018), such as IRS1 and IRS2. Both isoforms have a similar function; however, IRS1 may have a more significant role in normal glucose homeostasis. Dong et al. (2008) showed that liver‐specific Irs1 knockout animals presented considerable glucose intolerance, while Irs2 deletion resulted in mild glucose intolerance; deletion of both isoforms severely weakened insulin stimulation of PI3K‐Akt activity (Dong et al., 2008). The hepatic insulin signaling is distal to Akt activation; however, Akt signaling is central to hepatocellular insulin action. The Akt substrates are glycogen synthase kinase (GSK3), transcription factor forkhead box 01 (FOXO1), and mTORC1. Additional signaling pathways independent of Akt may be involved in metabolic control; however, more studies are needed to describe them (Lu et al., 2012).

In a fed state, insulin inhibits transcriptional gluconeogenic genes, especially those mediated by FOXO transcription factors. As previously mentioned, FOXO1 is an Akt target, and the main phosphorylation sites are at Thr24, Ser256, and Ser319. Mice lacking FOXO1 in their liver presented impaired hepatic glucose production (HGP) (Matsumoto et al., 2007). Therefore, FOXO1 is an important key molecule for HGP. Another critical role of insulin in the liver is its direct effect on lipid metabolism. Insulin regulates genes responsible for de novo lipogenesis (DNL), the conversion of sugar into fat ( Schwarz et al., 2017). The primary transcription factor regulator of this process is the sterol regulatory element‐binding protein‐1c (SREBP‐1c). Insulin regulates activation of SREBP‐1c; however, inhibition of the PI3K‐Akt‐mTORC1 axis is known to also inhibit SREBP‐1c via insulin inhibition (Li et al., 2010). Finally, insulin also regulates protein synthesis in the hepatocytes besides playing a role in glucose and lipid metabolism. The primary mediator of protein synthesis in various insulin‐responsive tissues, such as hepatocytes, adipocytes, and myocytes, is mTOR.

### 4.2 Insulin resistance

Defects of normal insulin function significantly contribute to the onset of hepatic insulin resistance and T2D. Studies suggest that FOXO1 dysregulation contributes to increased hepatic gluconeogenesis in humans with T2D (O‐Sullivan et al., 2015). FOXO1 increases enzyme production necessary for gluconeogenesis; therefore, upregulation of it results in increased substrate conversion into liver glucose (Czech, 2017). Obese mice with upregulation of Foxo1 expression became insulin insensitive. The precise mechanism of overfeeding in the dysregulation of Foxo1 is under investigation (Qu et al., 2006). However, the ablation of hepatic Foxo1 in mice demonstrated to improve increased gluconeogenic enzyme expression and normalize glucose tolerance (Dong et al., 2008). Recent evidence suggested that low hepatic PGC 1α in T2D patients is also associated with hepatic insulin resistance. PGC 1α drives the ratio of IRS1 and IRS2 in hepatocytes, and low levels of it resulted in disruption of IRS1 and 2 expression impacting normal glucose homeostasis (Besse‐Patin et al., 2017, 2019).

As insulin also plays a role in lipid metabolism via SREBP‐1C, as previously described, insulin‐resistant people may present decreased lipogenesis. As shown in animal models of hepatic insulin resistance, there is a decrease in hepatic DNL (Biddinger et al., 2008). Several effectors act together and are involved in insulin resistance, for instance, Akt and FOXO1, controlling both glucose and lipid handling (Manning & Toker, 2017). In a nonalcoholic fatty liver disease (NAFLD), increased re‐esterification of circulating fatty acids supplied by adipose insulin resistance is possibly one of the leading causes of increased liver triglyceride (Donnelly et al., 2005). Furthermore, in insulin‐resistant patients, the primary lipogenic flux is re‐esterification, and not DNL; DNL is regulated by SREBP‐1C, activated by mTORC1 via amino acid stimulation (Li et al., 2010).

As shown in Figure 2, ectopic lipid accumulation in muscle or liver is a consequence of an overfed state or defective adipocyte fatty acid metabolism. As a result, this leads to activation of DAG‐PKCε axis in the liver and subsequent inhibition of INSR signaling via phosphorylation of INSR at Thr1160 (Akhtar et al., 2019). Alternatively, ceramides have also been shown to activate an atypical PKCζ (Zeta) isoform and mediate hepatic insulin resistance (Xia et al., 2015). Therefore, some of the main consequences of this lipotoxicity‐induced insulin resistant state are impaired insulin stimulation of hepatic glycogen synthesis, impaired upregulation of DNL transcription genes, and impaired downregulation of gluconeogenic transcription genes. As noted above, the role of impaired mitochondria function in contributing to insulin resistance in the muscle is well established. Importantly, in the liver, insulin resistance originating from lipotoxicity has no link with impaired mitochondrial capacity, which has been attributed to a mitochondrial adaptation to promote increased lipolysis (Jelenik et al., 2017). More recent literature has highlighted the role of mitophagy in promoting mitochondrial fatty acid oxidation, as a consequence reducing hepatic fatty acid accumulation, leading to improved hepatic insulin resistance (Su et al., 2019). Studies showed that hepatic fatty acid accumulation resulted in an increase in the accumulation of damaged mitochondria (Wu et al., 2015); however, PINK1/Parking‐mediated mitophagy could reverse this phenomenon (Nguyen et al., 2016; Wang et al., 2019).

## 5 ADIPOSE

### 5.1 Insulin signaling

Adipose tissue is critically important in influencing both glucose and lipid metabolism (Kershaw & Flier, 2004; Scherer, 2006) by releasing adipokines, proinflammatory cytokines, and free fatty acids (FFAs) (Jung & Choi, 2014). Moreover adipose tissue is an insulin‐responsive tissue, whereby insulin prompts the storage of triglycerides by such methods as stimulating the differentiation of preadipocytes to adipocytes, inhibiting lipolysis, and increasing the uptake of fatty acids and glucose (Perry et al., 2015). Similar to the mechanisms in muscle, insulin exerts its biological effects via the IRS‐PI3K‐Akt2‐GLUT4 signaling pathways (Figure 2). However, both IRS1 and IRS2 are involved in adipocyte insulin signaling, in contrast with hepatocytes, where IRS1 has a more significant role in glucose homeostasis as compared to IRS2. Also, in a similar fashion as the skeletal muscle, Rab GAP TBC1D is expressed in adipocytes, though in lower levels, and contributes to the regulation of insulin signaling through vesicle trafficking and translocation of GLUT4 to the plasma membrane (Chadt et al., 2015).

As mentioned above, a major role of insulin in adipose tissue is to promote the suppression of lipolysis (Perry et al., 2015). Lipolysis is a process where lipid triglycerides are hydrolyzed into glycerol and fatty acids and used to provide stored energy during fasting or exercise. The mechanism that regulates lipolysis is highly dependent on the protein kinase A (PKA) signaling pathway. PKA phosphorylates the hormone‐sensitive lipase (HSL) and perilipin (PLIN) to promote lipolysis (Jaworski et al., 2007), where phosphodiesterase 3B (PDE3B) inhibits PKA by degrading cAMP (required for PKA activation). Consequently, PDE3B impedes the action of the pro‐lipolytic hormones HSL and PLIN, inhibiting lipolysis (Jaworski et al., 2007). In a fed state, insulin activates Akt2, which activates PDE3 and inhibits PKA, via unknown mechanism, thereby suppressing lipolysis (Figure 2). However, with adipose tissue insulin resistance, there is a decrease in Akt2 phosphorylation, resulting in sustained lipolysis activation (Morigny et al., 2016). As a result, non‐esterified fatty acid (NEFA) production and circulating fatty acids are increased, which are taken up by the liver and muscle, contributing to ectopic lipid accumulation in both tissues (Figure 2).

### 5.2 Insulin resistance

In the presence of an obesogenic environment, excess energy storage leads to the hypertrophy of adipocytes. Studies investigating the relationship between adipocyte size and adipokine secretion have demonstrated that adipocyte size affects the secretion of many adipokines (Skurk et al., 2007). Specifically, proinflammatory adipokines are significantly increased in large adipocytes compared to smaller ones (Skurk et al., 2007). Furthermore, TGFβ, a potent anti‐adipogenic inflammatory cytokine, is released from hypertrophic and dysfunctional adipocytes of obese mice and humans (Wu & Derynck, 2009). Therefore, a reduction in anti‐inflammatory adipokine secretion, concurrent with an increase in inflammatory cytokine secretion, may play an important role in the onset of adipose tissue insulin resistance (Ghaben & Scherer, 2019; Petersen & Shulman, 2018; Skurk et al., 2007).

Inflammatory neutrophil cells are the first to infiltrate WAT in a high‐fat feeding state and later recruits and activate the adipose tissue macrophages (ATM) (Soehnlein et al., 2008). Additionally, the mechanisms by which ATM activation leads to insulin resistance is dependent on cytokines activation. Inflammatory cytokines, such as tumor necrosis factor (TNF)‐α, interleukin‐1 beta and 6 (IL‐1β, IL‐6), are increased in obese diabetic humans and rodents (Kany et al., 2019), and neutralization of TNF‐α improves insulin sensitivity in obese rodents (De, 2000). Further, TNF‐α has been shown to induce serine phosphorylation of IRS‐1, thereby decreasing its association with PI3K and impeding insulin signaling (Rui et al., 2001). Additionally, the inflammatory mediators, TNF⍺ or IL‐1β increase lipolysis and inhibit INSR; therefore, impairing insulin signaling (Figure 2). These findings suggest that obesity‐induced insulin resistance may partially result from an imbalance in the secretion of pro‐ and anti‐inflammatory adipokines. A recent study suggest that TNF can contribute to insulin resistance as diet‐induced obesity triggers TNF‐dependent augmentation of circulating inflammatory monocytes independent of adiposity markers or expansion of adipose tissue (Breznik et al., 2018). Although the exact mechanisms remain unclear, an uncontrolled production and/or secretion of these cytokines from excess adipose tissue can lead to the development of insulin resistance and metabolic disease.

The suppression of lipolysis in conjunction with a decrease in the uptake of triglycerides, as observed in the presence of elevated insulin levels, can further add to the deleterious effects of ectopic lipid accumulation (Saponaro et al., 2015). Hyperinsulinemia, or excess secretion of insulin, is thought to cause insulin resistance, via unknown mechanisms (Johnson & Templeman, 2016). Hyperinsulinemia is associated with excess adiposity. Subsequently, dietary and pharmacological manipulations that reduce insulin may lead to a reduction in adipose tissue and greater insulin sensitivity, though the results of such interventions have been mixed (Alemzadeh et al., 1998; Due et al., 2007).

Recent studies suggest that significant events such as mitochondrial dysfunction and mitophagy are also involved in the development of insulin resistance adipose tissue (Wu et al., 2019). Evidence indicates that mitochondrial content and mitochondrial oxidative capacity are altered in several insulin‐responsive tissues (such as adipose tissue), in humans and animal models presenting with obesity and insulin resistance (Jeong‐a et al., 2008). It was identified that Fundc1 acts as an important mitophagy receptor in adipocytes by mediating mitophagy through its interaction with MAP1LC3B in response to hypoxia (Liu et al., 2012), and a deficiency in this receptor is linked to insulin insensitivity and metabolic disorders (Wu et al., 2019). Notably, abnormal Fundc1‐ mediated mitophagy in adipose tissue resulted in increased oxidative stress and hyperactivation of MAPK signaling, giving rise to ATMs infiltration and sustained inflammatory response (Wu et al., 2019).

Although evidence suggests a link between mitochondrial dysfunction and insulin resistance, dysfunctional mitochondria may not be necessary to induce insulin resistance in adipocytes. Recently, a study investigating the role of adipose inflammation, mitochondrial dysfunction, and gut dysbiosis in obesity‐induced insulin resistance, the authors found that mitochondrial dysfunction and gut dysbiosis occurred during HFD‐feeding but was not present in spontaneously obese mice (Petrick et al., 2020). This suggests that mitochondrial dysfunction may be diet‐related and is not always required for obesity‐induced insulin resistance.

Certainly, adipose tissue is an essential regulator of overall health. Therefore, impaired adipose tissue function may lead to a series of severe global health complications such as insulin resistance, T2D, among other metabolic disease (Britton et al., 2013; Marinou et al., 2014; Silva et al., 2017).

## 6 NOVEL MECHANISMS OF CROSS‐TALK BETWEEN MUSCLE, ADIPOSE, AND LIVER

Classically, integrative biochemistry has focused on the provision of metabolic fuels needed by the liver to maintain a glucose supply for the brain during times of stress, starvation, disease, or muscular exertion. Fundamentally, this cross‐talk between muscle, adipose, and liver is regulated by insulin, glucagon, and the traditional counter‐regulatory hormones, such as glucocorticoids, adrenaline, and growth hormone. Key examples of this cross‐talk include the Cori cycle (lactate‐glucose cycle), the Cahill cycle (glucose‐alanine cycle), as well as gluconeogenic consumption of glycerol and glutamine (Sharma et al., 2019; Xu et al., 2011). However, research over the past few decades have identified the gut, and the production of GLP‐1 and GIP as fundamental modulators of not only insulin secretion, and the crosstalk between muscle, adipose, and liver, but also as regulators of appetite by activating receptors in the lateral hypothalamus, other regions of the diencephalon, and brainstem (Müller et al., 2019). More recently, other growth factors, along with inflammatory and innate immunity mediators, have been shown to be released from muscle, adipose, liver, and gut tissues as a means to directly communicate metabolic cues between the classical insulin sensitive tissues.

Many mechanistic studies examining the relationship between obesity and its associated chronic low‐grade inflammation have highlighted the role of pattern recognition receptors (PPRs), specifically Toll‐like receptors (TLRs) and Nucleotide‐binding oligomerization domain (NOD)‐like receptors or (NLRs), and their impact on insulin resistance (Petrick et al., 2020; Schertzer et al., 2011). Obesity is thought to provoke increased intestinal permeability, giving rise to higher circulating levels of lipopolysaccharide (LPS) emitted by intestinal gram‐negative bacterial species (Saad et al., 2016). This, in turn, may initiate an inflammatory cascade via activation of PRRs, such as TLR4 (member of the Toll‐like receptor family) in adipocytes (Kawasaki & Kawai, 2014). The binding of LPSs or FFAs to TLRs promotes the downstream signaling of the transcription factor, nuclear factor kappa‐light‐chain‐enhancer of activated B cells (NF‐κB), which regulates the expression of pro‐inflammatory cytokines and chemokines (Rogero & Calder, 2018). Many of these inflammatory proteins work to activate serine kinases, such as JNK, which directly blocks insulin action in muscle, liver and adipose tissue (Solinas & Becattini, 2016); conversely, selective inhibition of JNK in adipose tissue has been demonstrated to protect against diet‐induced obesity, improving insulin sensitivity in rodent liver and skeletal muscle (Zhang et al., 2011). Similarly, the NLR family of PRRs also responds to obesity‐induced signals, such as damage‐associated molecular patterns (DAMPs), derived from stressed adipocytes (Jin & Flavell, 2013). Moreover, acute activation of NOD proteins has been demonstrated to induce whole‐body insulin resistance in mouse models (Schertzer et al., 2011), which further supports the link between innate immune signaling and whole‐body metabolism.

The growth differentiation factor 15 (GDF15) is a stress‐induced protein member of the TGFβ superfamily of proteins, increased in many disease states (Tsai et al., 2016). While various tissues ubiquitously express GDF15 in physiological levels, it becomes selective induced upon nutrient challenge. Patel et al., (2019) have recently demonstrated that GDF15 is selectively upregulated as a stress response mechanism during high‐fat feeding. In their study, animals treated with a HFD presented with high GDF15 mRNA expression levels in metabolic tissues such as liver and adipose (brown and white); however, very little was detected in the skeletal muscle (Patel et al., 2019). Furthermore, the increase in GDF15 caused an aversive endocrine signal in the brain, which may have contributed to weight loss, therefore representing a potential role in obesity therapy (Patel et al., 2019). Day et al. (2019) have recently elucidated a possible mechanism by which GDF15 can suppress appetite and promote weight loss. In their study, metformin augmented GDF15 secretion in primary hepatocytes via increased upregulation of the activating transcription factor 4 (ATF4) and C/EBP homologous protein (CHOP). In a rodent model exposed to HFD, metformin increased serum levels of GDF15 concurrent with reductions in food intake, body mass, fasting insulin and improved glucose intolerance. Even though it was not clear the tissue sources of GDF15 produced in response to metformin in vivo, they also observed that an increase in GDF15 correlated with weight loss in T2D patients under metformin treatment (Day et al., 2019).

GDF15 is also known as a mitochondrial disorder biomarker, however, the precise pathological mechanism is not very clear. Ost and colleagues (2020) used transgenic‐mice harboring deficient muscle‐specific mitochondrial OXPHOS capacity via respiratory uncoupling (Ucp1‐TG). These animals presented specific induction of GDF15 in skeletal muscle with diurnal variation (Ost et al., 2020). Interestingly, Ucp1‐TG combined with GDF15 knockout showed progressive weight gain, concurrent with muscle mitochondrial stress‐induced metabolic inflexibility, insulin insensitivity, and impaired browning of WAT (Ost et al., 2020). These new findings collectively represent novel pathophysiological roles of mitochondrial stress in the induction of GDF15 and the regulation of systemic energy metabolism.

The exact mechanisms of muscle mitochondrial quality control and its effects are currently an emerging area of investigation; however, not yet well elucidated. A novel study by Fu et al. (2018) described the important role of Fundc1, a mitophagy protein, in attenuating diet‐induced obesity and its cross‐talk effects in muscle and adipose tissue. Using two skeletal muscle‐specific Fundc1 knockout models, they demonstrate that mice lacking Fundc1 exposed to HFD had impaired LC3‐mediated mitophagy and mitochondrial energetics. However, these animals were protected against HFD‐induced obesity and insulin resistance. Furthermore, in a compensatory mechanism in the absence of Fundc1, it led to an activation of adipose tissue adaptive thermogenesis via secretion of fibroblast growth factor (FGF) 21 from muscle (Fu et al., 2018). These findings suggest that the muscle mitophagy response not only regulates muscle insulin sensitivity, but can modulate whole body metabolism via FGF21 secretion.

FGF21 is another critical therapeutic target on the rise that promotes protection from lipid‐induced muscle and liver insulin resistance and T2D (Camporez et al., 2013). FGF19, FGF21, and FGF23, are essential in the regulation of glucose and lipid metabolism, as well as the whole‐body homeostasis (Degirolamo et al., 2016). Most importantly, FGF21 plays a role as a hepatokine, adipokine, and myokine controlling insulin sensitivity in insulin‐resistant animals. Furthermore, FGF21 also improves fat oxidation in the muscle, DNL in the liver, and thermogenesis in BAT and WAT (Coskun et al., 2008; Klein Hazebroek & Keipert, 2020). In an animal study, mice treated with FGF21 had decreased lipid accumulation, such as diacylglycerol in both muscle and liver, concurrent with reduced activation of PKC proteins. The precise mechanisms of how FGF21 regulates insulin signaling are to date, not entirely known.

Recently, Fu et al. (2018) demonstrated that FGF21 was activated via a retrograde activation by ATF4, where the latter was increased in myotubes of Fundc1 muscle knockouts. The role of ATF4 on insulin metabolism has been highly investigated over the years, where it has been previously shown to be a negative regulator of insulin secretion and sensitivity to insulin in the liver, muscle and fat (Yoshizawa et al., 2009). Specifically, in the liver, Zhang and colleagues (2013) demonstrated that hepatic insulin resistance was mediated by the ATF4/mTOR/S6K1 axis (Zhang et al., 2013). In a parallel study by Pereira and colleagues (2017) describing the role of mitochondrial dynamics in muscle insulin sensitivity demonstrated that OPA1 deficiency‐induced mitochondrial dysfunction and triggered ER stress and concurrent activation FGF21 (Pereira et al., 2017). These observations collectively highlight a conserved mechanism where both mitochondrial stress and ER‐stress, likely activated by the UPR regulators ATF4, ATF5, and CHOP, control the secretion of FGF21 and GDF15 in insulin sensitive tissues to orchestrate a metabolic cross‐talk leading whole body nutrient homeostasis including appetite control.

## 7 SUMMARY AND CONCLUSION

The present review identified a plethora of studies that identify mechanisms of insulin resistance in muscle, adipose, and liver tissue. Additionally, it outlines important differences in insulin signaling and the development of insulin resistance in these tissues. Normal insulin functioning is essential for skeletal muscle's energy expenditure and glucose metabolism. However, disruption of INSR‐ IRS1‐PI3K‐Akt axis compromises normal insulin action in the muscle and leads to insulin resistance. Similarly, in adipose and liver tissue, disruption of the insulin signaling IRS‐PI3K‐Akt axis leads to severe glucose intolerance and insulin resistance, as described by many INSR, IRS, and Akt knockout studies investigating their effects on proximal insulin signaling and body glucose homeostasis (Tables 1 and 2).

**TABLE 1:** Phenotypes of INSR and IRS knockouts

Receptor	Tissue	Specie	Phenotype	References
Insr	Whole‐body	Mice	Drastic hyperglycemia and hyperketonemia. Death shortly after birth due to Ketoacidosis	PubChem
Insr	Whole‐body	Mice	Early postnatal death	Okamoto et al. (2004)
Insr	White and Brown adipose tissue	Mice	Impaired insulin mediated GLUT4 translocation and lipolysis suppression; protected from glucose intolerance	Blüher et al., (2003; Blüher et al., 2002)
Insr	Muscle	Mice	Impaired insulin‐stimulated glucose uptake and muscle glycogen synthesis	Kim et al., (2000)
Insr	Liver	Mice	Impaired insulin signalling and suppression of HGP; severe glucose intolerance	Michael et al. (2000)
Insr	Muscle	Mice	Increased fatty mass, serum triglycerides, and free fatty acids; normal glucose tolerance	Brüning et al., 1998)
Irs1	Whole‐body	Mice	Growth retardation; reduced insulin sensitivity, though not associated with T2D; hyperinsulinemia	Boucher et al. (2014)
Irs1 and Irs2	Whole‐body	Mice	No diabetic phenotype in neither whole‐body nor muscle specific IRS1 and IRS2 knockouts	Long et al. (2011)
Irs1	Liver	Mice	Severe glucose intolerance	Dong et al. (2008)
Irs1	Whole‐body/ WAT	Mice	Insulin resistance and activation of a growth‐related pathway exclusively in white adipose tissue.	Araújo et al. (2005)
Irs1	Whole‐body	Mice	Delayed embryonal and postnatal growth; insulin resistance and low glucose‐lowering effects	Tamemoto et al. (1994)
Irs2	Liver	Mice	Mild glucose intolerance	Dong et al. (2008)
Irs2	Whole‐body	Mice	Obesity; reduced insulin sensitivity; severely impaired glucose tolerance; hyperinsulinemia	Masaki et al. (2004)
Irs2	Muscle	Mice	Normal dose‐dependent insulin‐stimulated glucose uptake	Higaki et al. (1999)
Irs2	Whole‐body	Mice	Peripheral insulin resistance (muscle, liver) and impaired β‐cell function	Withers et al. (1998)

**TABLE 2:** Phenotypes of Akt knockouts

Receptor	Tissue	Specie	Phenotype	References
Akt1/Akt2	Whole Body	Mice	Muscle and body mass loss concurrent with reduction in TBC1D1	Jaiswal et al. (2019)
Akt1	Whole Body	Mice	Deletion in Akt2 null mice, resulted in severe hyperglycemia	Lu et al. (2012)
Akt1	Skeletal Muscle	Mice	Increased expression of two autophagic genes (Bnip3 and garabap1)	Reynolds et al. (2012)
Akt1	Whole Body	Mice	Weighed significantly less and had 15% less lean mass than wild type	Goncalves et al. (2010)
Akt1	Skeletal Muscle	Mice	Significant decrease in mass compared to wild type in the following muscles: extensor digitorum longus (EDL), gastrocnemius, anterior	Goncalves et al. (2010)
Akt2	BAT	Mice	Targeted adipocyte lineages generated smaller adipocytes that correlated with the appearance of tissue atrophy. Non‐targeted lineages on the other hand showed hypertrophy. Essentially, a redistribution of fat	Sanchez‐Gurmaches et al. (2019)
Akt2	Whole Body	Mice	Mildly diabetic	Lu et al. (2012)
Akt2	Whole Body	Mice	Weighed significantly less and had a 44% decrease in fat mass compared to wild type	Goncalves et al. (2010)
Akt2	Skeletal Muscle	Mice	Decrease in the mass compared to WT in gastrocnemius and EDL, but a 21% increase in soleus mass.	Goncalves et al. (2010)
Akt2	Hepatic	Mice	Decreased liver weight, de novo lipogenesis, and triglyceride levels; Inhibition of steatosis	Leavens et al. (2009)

Previous studies in both humans and rodents have established that lipid‐induced insulin resistance in hepatic and skeletal muscle tissue are both dependent on the DAG‐PKC axis, resulting in the phosphorylation of either IRS1 or IRS2 (Mizushima, 2018). Like the muscle and liver, adipose tissue insulin resistance plays an important role in the development of T2D (Goedeke et al., 2019). Studies have suggested that disproportionate secretion of pro‐ and anti‐inflammatory adipokines and reduction in lipolysis is concurrent with insulin resistance in adipose tissue. Specifically, impaired insulin suppression of lipolysis in adipose tissue leads to increased circulating plasma fatty acid and uptake by liver and muscle tissue leading to lipotoxic intracellular environments. These cascades ultimately prevent the translocation of GLUT4 receptors to the membrane surface of skeletal muscles and adipose tissue.

In addition, other factors, including metabolic inflexibility, mitochondrial dysfunction, mitophagy, and ER stress have all been linked to the pathogenesis of insulin resistance (Figure 1). As previously described, metabolic inflexibility is the inability to switch fuel sources that can consequently increase lipid levels and contribute to insulin resistance. Mitochondrial dysfunction, meanwhile, describes an increase in ROS that induces oxidative damage to the mitochondria, leads to lipid accumulation, and likely involves the mitochondrial/ER UPR in the regulation of FGF21 and GDF15 secretion. Furthermore, induced oxidative damage elicits a mitophagy response to remove damaged mitochondria, while lysosomal recruitment to mitochondria can activate mTOR‐S6K signaling to inhibit IRS1 (Figure 1). Taken as a whole, these mechanisms providing compelling evidence to the central role of lipid toxicity in insulin resistance and the downstream mechanisms are indeed intricate and interconnected. Interestingly, mitophagy genes, such as Nix, Bnip3, and Fundc1, have also been associated with lipid metabolism. Moreover our group recently observed that Nix is elevated in insulin‐resistant muscle, and serves as a central regulator of mitochondrial‐ER stress, mitophagy, and lysosomal signaling leading to an impaired insulin response.

Finally, current animal and human research are highlighting the importance of tissue crosstalk in the regulation of lipid‐induced insulin resistance, and the emerging role of secreted growth factors and the innate immune response as important mechanisms orchestrating target tissue insulin sensitivity, summarized as an illustration (Figure 2). It is also evident that each peripheral tissue responds in a distinct and cell‐type specific manner to impact whole‐body metabolism. Hence, there is a necessity for further research to fill important knowledge gaps in the pathogenesis of insulin resistance using tissue‐specific approaches that also consider inter‐tissue cross‐talk and whole organism metabolism (Figure 1). It is only with a concrete understanding of the unique responses of each tissue during insulin resistance and hyperinsulinemia that effective therapeutic strategies can be developed to provide a better quality of life for those affected by these metabolic conditions.

## 8 ETHICS

Not applicable.

## CONFLICT OF INTEREST

The authors do not have any conflicts of interest to declare.
